# Age and menarcheal status do not influence metabolic response to aerobic training in overweight girls

**DOI:** 10.1186/1758-5996-5-7

**Published:** 2013-02-25

**Authors:** Neiva Leite, Humberto M Carvalho, Cristina Padez, Wendell Arthur Lopes, Gerusa E Milano, Rosana B Radominski, Manuel J Coelho-e-Silva

**Affiliations:** 1 Department of Physical Education, Federal University of Paraná, Curitiba, Brazil; 2 Faculty of Sports Sciences and Physical Education, University of Coimbra, Coimbra, Portugal; 3 Department of Life Sciences, University of Coimbra, Coimbra, Portugal; 4 Faculty of Physical Education, State University of Campinas, Campinas, Brazil; 5 Pediatric Endocrinology Unit, Department of Nutrition, Federal University of Paraná, Curitiba, Brazil; 6 Departamento de Educação Física, Universidade Federal do Paraná, Setor de Ciências Biológicas, BR 116, km 95, nº 19031, Jardim Botânico, CEP 81690-100, Curitiba, Paraná, Brazil

**Keywords:** Overweight, Multidisciplinary intervention, Maturation, Exercise

## Abstract

**Background:**

Multidisciplinary intervention is an alternative for the treatment of children and adolescent obese. However, the influence of age and menarcheal status in the pattern of metabolic response of obese girls has not been investigated. The following study examined the effects of a 12-week multidisciplinary intervention on metabolic health in overweight girls and the contribution of age and menarcheal status on the resulting changes.

**Methods:**

Eighty-eight overweight girls (10 - 16 years) were considered initially for this study and randomly assigned (intervention group: n = 58; control group: n = 30). Forty-six girls completed the intervention program and 16 girls completed the follow-up for the control group. The 12-week intervention included aerobic exercises (three times per week) and nutritional intervention. Anthropometrical measures (body mass, body mass index and waist circumference), menarcheal status and metabolic profiles including glucose, insulin, triglycerides (TG), total cholesterol (TC), high-density lipoprotein cholesterol (HDL-C) and low-density lipoprotein cholesterol (LDL-C) were assessed in the beginning and after of intervention. Additionally, were calculated homeostatic model assessment-insulin resistance (HOMA-IR) and quantitative insulin sensitivity check index (QUICKI).

**Results:**

After 12-week, girls decreased significantly the body mass (76.6 ± 14.7 to 75.7 ± 14.6 kg) body mass index (30.1 ± 4.0 to 29.4 ± 4.0 kg/m^2^) and waist circumference (98.9 ± 10.9 to 96.5 ± 11.4 cm). There were differences in HDL-C (43.1 ± 8.2 to 50.3 ± 9.4 mg/dl), TG (120.9 ± 64.3 to 93.3 ± 47.9 mg/dl) and insulin (16.9 ± 7.6 to 15.6 ± 9.8 mg/dl). Relative contribution of age was significant only for within-subject variability in waist circumference.

**Conclusions:**

The multidisciplinary based on aerobic training intervention used in this study produced substantial benefits on metabolic health indicators in overweight girls. The changes observed were not related to inter-individual variability in age and maturity status.

## Background

Overweight in childhood and adolescence is associated with increased risk of adult obesity and metabolic dysfunction as dyslipidemia, hypertension and insulin resistance. These conditions are involved in the atherosclerotic process and contribute to the development of cardiovascular diseases (CVD) [1,2]. Multidisciplinary interventions that provide young individuals with information about health and nutrition and promote active living have been proposed as attractive strategies for encouraging weight loss and reverting/preventing metabolic alterations in children and adolescents [3,4]. Particularly, exercise and nutritional guidelines have been used as therapeutic actions to reduce childhood obesity [5,6], modulate insulin sensibility [4,7] and improve cardiovascular protection [8].

The pattern of CVD markers response to multidisciplinary interventions in obese adolescents is not clear. There is evidence that age and advanced sexual maturity status affect lipids, glucose and insulin levels [9], as well as blood pressure [10]. When considering adolescent girls, changes associated to body size during pubertal growth show a trend to increase fat mass percentage, as sexual maturation (a late pubertal maturation event) is characterized by a myriad of hormonal changes [11]. Thus, the interpretation of the effects of a multidisciplinary treatment programs in obese adolescent girls on body mass should consider the possible inter-individual variability associated to biological maturation. In addition, there is little information about the effects of multidisciplinary treatment programs, involving physical activity and nutritional intervention in obese adolescent girls, on body size, body composition and metabolic markers [4,9,10], and its association with age and menarcheal status.

The purpose of this study was to determine the effects of a 12-week multidisciplinary intervention, based on aerobic training, on metabolic markers of overweight girls, and verify whether such changes induced by training are affected by age and menarcheal status.

## Methods

### Participants and study design

**Figure 1:**
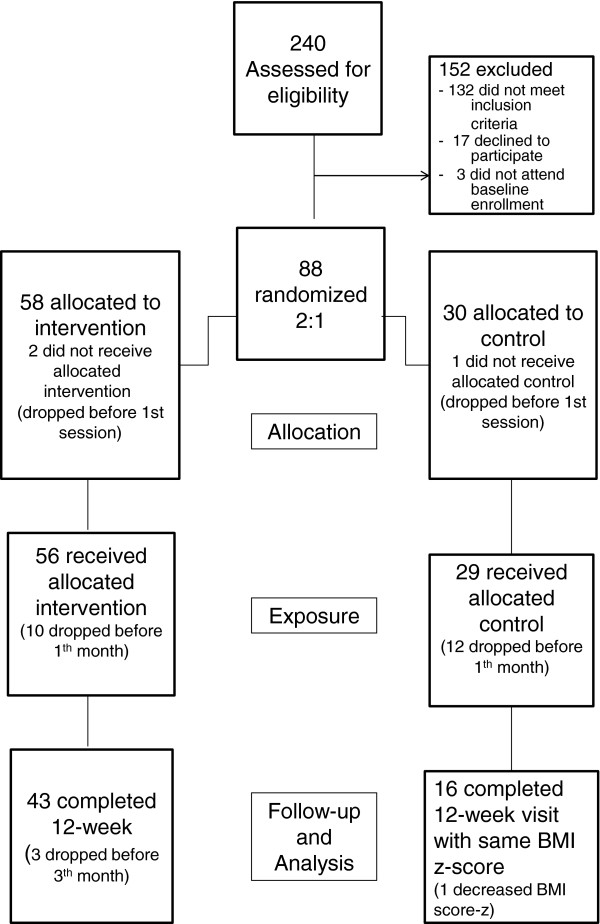
A schematic diagram indicating the flow of study subject selection though the study and subject compliance.

This study employed a pre-post controlled trial. Overweight girls aged between 10–16 years-old volunteered to participate in this study and were considered for analysis. The participants were recruited from the Pediatric Endocrinology Ambulatory and public schools of Curitiba, Paraná (Brazil). Participants were randomly assigned in a ratio of 2:1 [12]; multidisciplinary intervention group (n = 55) and control group (n = 30). A schematic map of the study design is shown in Figure 1.

The inclusion criteria were: girls aged 10- 16 years-old, with body mass index (BMI) above 85^th^ percentile [13] and sedentary for at least 4 months prior to study participation. Exclusion criteria were: presence of diabetes and the use of medication that could alter blood pressure, glucose or lipid metabolism; previous drug used such glucocorticoids, insulin sensitizers, or psychotropic medication which may affect appetite regulation; glucose levels above the limits for glucose intolerance [14]; orthopedic limitations and pregnancy. None of the subjects were taking regular medication, nor did they display any clinical manifestations of illness. The intervention group was exposed to a 12-week program based on aerobic exercise, nutritional program and educational meetings for the management of obesity. Control group participants were exposed to the same nutritional program as the intervention group and instructed about other lifestyle changes during their regular visits to the hospital, but did not participate on the exercise sessions as for the intervention group. Control group participants maintained their usual levels of daily activity, without additional exercise components. For the control group, exclusion criteria included not to gain body mass during the intervention period, in order to remove influence of body mass gain on the metabolic health indicators (-0.2 < BMI z-score < 0.2). Only 16 subjects were retained for the analysis in this group (36%). Meanwhile, two subjects were removed from the intervention group because their glucose levels were above the limits for glucose intolerance. Once placed in the groups, subjects were measured one week before and after the 12-week aerobic training based intervention. All participants and their families received information about the protocol and signed an informed consent form.

The study was approved by the Human Ethics Committee of the Federal University of Paraná (Protocol number CEP/HC 2460.067/2011-03).

### Anthropometry

All measurements were taken by a single experienced observer following standardized procedures [15]. Height was measured by a portable stadiometer (Ayrton Corporation, Prior Lake, Minnesota, USA) to the nearest 0.1 cm. Body mass (BM) was measured with the use of a portable balance (Filizola, São Paulo, Brazil) to the nearest 0.1 kg. Body mass index (BMI) was calculated as the weight in kilograms divided by the square of the height in meters. BMI was converted to z-score by subtracting the value corresponding to the 50^th^ percentile of BMI, divided by the standard deviation of the population [13]. Waist circumference was measured in centimeters using a non-extendable flexible tape, with precision of 0.1 cm. The tape was applied above the iliac crest, parallel to the ground, with the individual standing with the abdomen relaxed, arms along the body and feet together.

### Biological maturity status

All the participant girls were interviewed at clinical examination to obtain whether menarche occurred or not, in order to classify girls by menarcheal status. This indicator refers to a specific maturational event of sexual maturation that is limited to girls.

### Metabolic health assessment

Blood samples were obtained after a 12 h overnight fasting were used for measurements of plasma glucose, triglycerides (TG), total cholesterol (TC) and high-density lipoprotein cholesterol (HDL-C). CT, TG e HDL-c, and were evaluated by enzymatic colorimetric method (CHOD-PAP) (Merk, Darmstadt, German; Roche, Indianapolis, IN, EUA). Low-density lipoprotein cholesterol (LDL-C) was calculated using Friedewald’s formula [16]. The glucose was measured by enzymatic method (Glucose Oxidase – Labtest). The insulin was assayed by quimioluminescence (Immulite).

Insulin resistance was estimated through homeostatic model assessment-insulin resistance (HOMA-IR). Additionally, quantitative insulin sensitivity check index (QUICKI) was calculated [17].

### Intervention design

The intervention consisted of aerobic exercise, a nutritional program and educational meetings for the management of obesity. The group of professionals involved in the program included a physiologist, a nutritionist, six physical educators, two physicians and three nurses. The intervention took place in a clinic in the central region of Curitiba-Pr. Only the walks were made in a square nearby.

#### Exercise

All participants were required to attend exercise sessions two to three times per week during three months or 12 weeks. Exercise consisted of 45 minutes indoor cycling, 45 minutes outdoor walking/running and 20 minutes stretching, which were conducted by a certified fitness instructor. Exercise was prescribed for each participant, based on the data collected from the baseline treadmill and cycle ergometer test. During the first four weeks, intensity was set as 35–55% of heart rate reserve and was increased to 55–75% during the final eight weeks. Heart rate was monitored and registered every 15 minutes during all sessions. Instructors encouraged and helped participants to increase exercise intensity to maintain the heart rate within the target zones [18].

#### Nutritional Intervention and Educational Meetings

The nutritional intervention involved a qualitative and quantitative analysis of the subjects’ food intake based on a 3 day food record, two days per week and one day on the weekend. The Participant’s diet control (dietary recall) was taken in three days, according to the American Dietetic Association [19]. The diet emphasized the consumption of abundant vegetables, fresh fruit, regular consumption of dairy products (principally cheese and yogurt), fish and poultry consumed in low to moderate amounts, and a reduced intake of red meat. This diet has been specifically devised for adolescents by our nutritionist. Total fat in this diet is 25% to 35% of the total caloric intake. Families were provided with additional nutritional instruction, including interpretation of food labels and shopping, and were taught stimulus control to reduce access to high-calorie foods and increase access to healthy lower-calorie foods. All participants were encouraged to maintain an active lifestyle during and after the multidisciplinary program. The adolescents participated in educational meetings once-per month during 60 minutes. The meetings were taught by physical educators and nutritionist focused on daily diet, physical activities and reduction of sedentary behavior, such as watching television or playing computer games.

### Statistical analysis

All dependent variables were log-transformed before analysis to reduce non-uniformity of error and to express effects as percent changes, except BMI that was standardized calculating z-scores. Descriptive statistics of all measures are presented as mean ± standard deviation. Changes in metabolic health indicators as a consequence of training were examined based on paired-t statistics (expressed effects as percent changes). Expressed as coefficients of variation, within-individual variation represents typical variation in an subject’s measure scores; for typical variation in an subject’s change score between two measures, the typical variation needs to be multiplied by the square root of 2 [20]. To make inferences about the true (population) values of the effect of training on metabolic health, the 90% confidence limit for each effect was also calculated (CL) [21]. An effect was considered unclear if its confidence interval overlapped substantially positive and negative values, if the effect is beneficial or harmful [21]. The between-subject standard deviation for each dependent variable was used to convert the log-transformed changes in performance into standardized [Cohen effect size (ES)] changes in the mean. The smallest standardized change was assumed to be 0.20 [22].

Additionally, the influence of inter-individual variation in chronological age and menarcheal status (dummy variable: 0 for menarche has not occurred; 1 for menarche has occurred) on changes with training were modeled using a proportional, curvilinear model [23]. Significance was set at *p* < 0.05. Statistical analyses were performed using SPSS version 17.0 software (SPSS, Chicago, IL).

## Results

Characteristics of intervention and control groups at baseline are presented in Table 1. In the intervention group, 17 girls had not attained menarche (11.3 ± 1.2 years) and 26 girls had already passed menarche (14.2 ± 1.2 years). There were no differences in anthropometric variables, while some metabolic variables presented differences between the groups.

**Table 1:** Characteristics of intervention and control groups at baseline

	**Intervention group (n = 43)**	**Control group (n = 16)**	***p***
Chronological age (years)	13.1 (1.9)	12.4 (1.5)	0.20
Height (cm)	158.9 (8.2)	156.3 (8.9)	0.34
Body mass (kg)	76.6 (14.7)	74.8 (21.7)	0.77
Body mass index (kg/m^2^)	30.1 (4.0)	30.2 (6.2)	0.98
Body mass index (z-score)	3.0 (1.1)	2.7 (0.8)	0.27
Waist circumference (cm)	98.9 (10.9)	96.4 (14.2)	0.53
Total cholesterol (mg/dl)	159.3 (31.2)	160.1 (27.7)	0.93
HDL-C (mg/dl)	43.1 (8.2)	42.6 (4.7)	0.79
LDL-C (mg/dl)	92.4 (26.0)	91.8 (25.1)	0.93
TG (mg/dl)	120.9 (64.3)	129.3 (56.0)	0.63
Glucose (mg/dl)	87.5 (7.2)	93.5 (4.9)	0.00
Insulin (μIU/ml)	16.9 (7.6)	23.9 (5.2)	0.00
HOMA-IR	3.691 (1.750)	5.533 (1.279)	0.00
QUICKI	0.32 (0.03)	0.30 (0.01)	0.00

Table 2 shows the effects of the training program in the intervention group. There were no differences in height, body mass and BMI during the 12-week training. The trend of changes in body size as consequence of training seems to be possibly beneficial in the waist circumference and BMI z-score, which considers age. The differences in body dimensions before training were probably trivial between subjects from both the intervention group and control group. Changes in body dimensions between groups, as consequence of training, were apparent in BMI standardized (-9.3%, *p* < 0.01, possibly beneficial) and waist circumference (-4.2%, *p* < 0.01, probably beneficial).

**Table 2:** Mean changes in metabolic health indicators pre- and post-training in the intervention group and chances that the true difference in the changes is substantial (n = 43)

	**Pre-training**	**Post-training**	**Changes in mean, 90% CL (%)**	**Coefficient of variation, 90% CL (%)**	**Practical inference**
Height (cm)	158.9 (8.2)	159.9 (8.0)	0.7 (0.5 to 0.9) *	0.6 (0.5 to 0.7)	Probably trivial
Body mass (kg)	76.6 (14.7)	75.7 (14.6)	-1.1 (-2.0 to -0.3) *	2.3 (2.0 to 2.8)	Probably trivial
BMI (kg/m^2^)	30.1 (4.0)	29.4 (4.0)	-2.5 (-3.2 to -1.7) *	2.1 (1.8 to 2.6)	Probably trivial
BMI (z-score)	3.0 (1.1)	2.7 (1.1)	-10.1 (-12.4 to 7.8) *	7.2 (6.1 to 8.9)	Benefit possible
WC (cm)	98.9 (10.9)	96.5 (11.4)	-2.5 (-3.4 to -1.6) *	2.7 (2.3 to 3.3)	Benefit possible
Total CT (mg/dl)	159.3 (31.2)	159.6 (31.6)	0.3 (-2.5 to 3.2)	8.2 (6.9 to 10.1)	Possibly trivial
HDL-C (mg/dl)	43.1 (8.2)	50.3 (9.4)	17.0 (11.9 to 22.3) *	13.0 (10.9 to 16.1)	Benefit likely
LDL-C (mg/dl)	92.4 (26.0)	91.0 (24.5)	-0.4 (-5.9 to 5.4)	16.8 (14.1 to 20.9)	Unclear
TG (mg/dl)	120.9 (64.3)	93.3 (47.9)	-22.7 (-29.6 to -15.1) *	29.5 (24.6 to 37.1)	Benefit possible
Glucose (mg/dl)	87.5 (7.2)	88.8 (6.8)	1.6 (-0.9 to 4.3)	7.3 (6.1 to 8.9)	Possibly trivial
Insulin (μIU/ml)	16.9 (7.6)	15.6 (9.8)	-14.1 (-24.1 to – 2.9) **	40.6 (33.6 to 51.6)	Benefit possible
HOMA-IR	3.691 (1.750)	3.412 (2.121)	-12.7 (-22.9 to -1.2)	40.8 (33.7 to 51.8)	Benefit possible
QUICKI	0.32 (0.03)	0.33 (0.03)	2.0 (0.3 to 3.9)	5.0 (4.2 to 6.2)	Benefit possible

* p < 0.01; ** p < 0.05, comparisons between pre- and post-training based on paired-t statistic.

**Figure 2:**
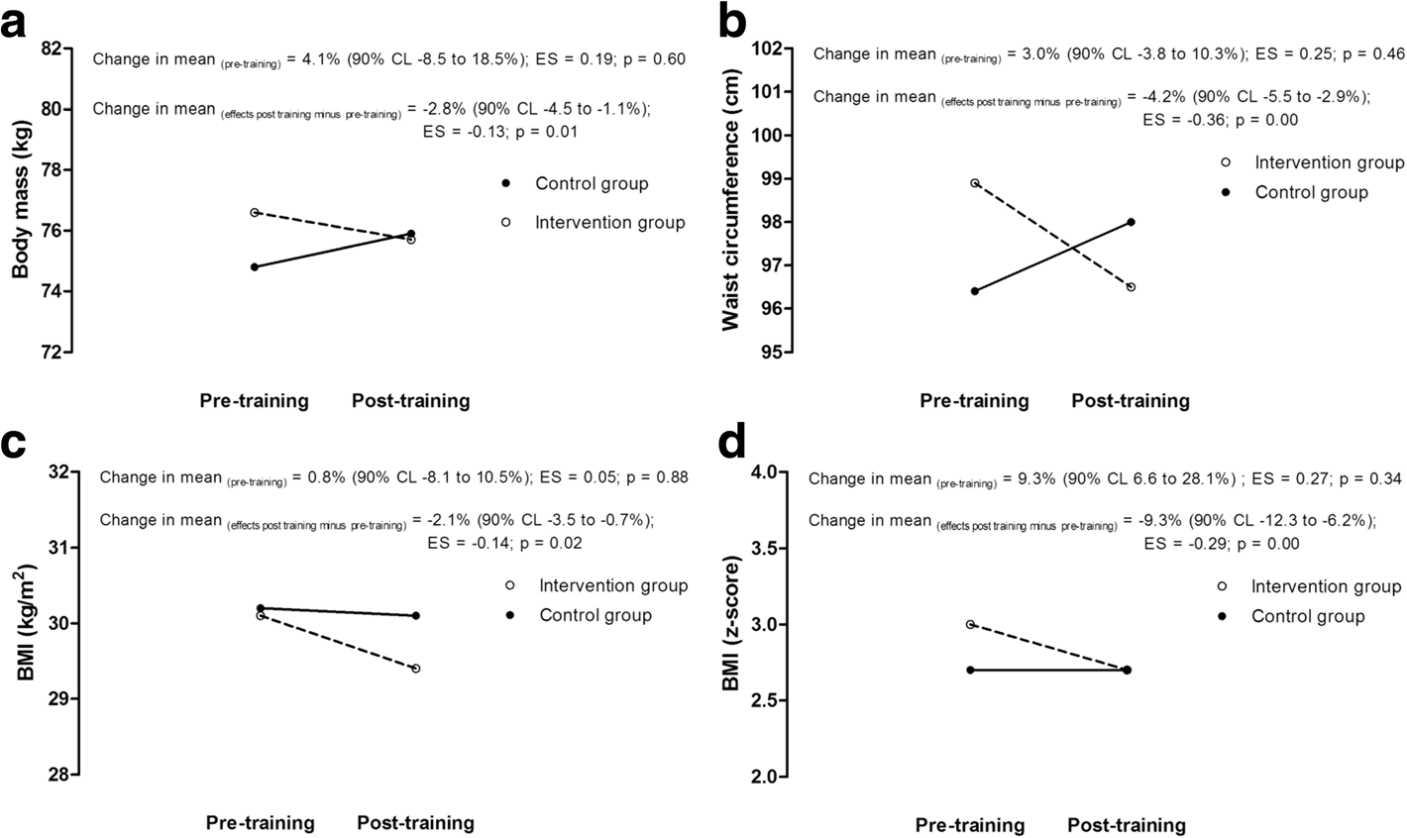
**Mean changes in anthropometric variables pre- and post-training in the intervention group and control group.** (**a**. body mass; **b**. waist circumference; **c**. BMI; **d**. BMI z-score).

**Figure 3:**
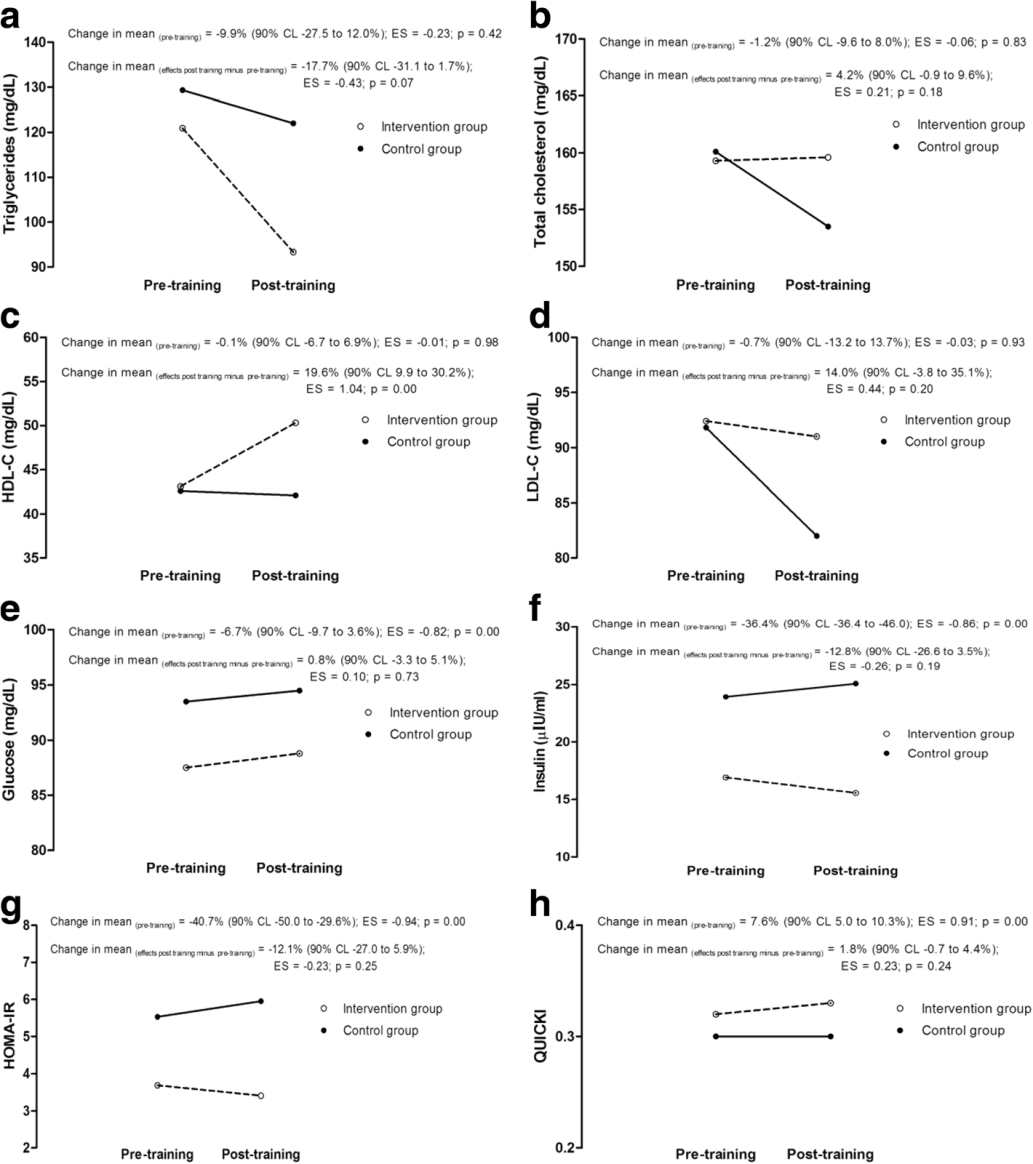
**Mean changes in metabolic health indicators pre- and post-training in the intervention group and control group.** (**a**. triglycerides; **b**. total cholesterol; **c**. HDL-C; **d**. LDL-C; **e**. glucose; **f**. insulin; **g**. HOMA-IR; **h**. QUICKI).

The individual responses for the intervention group in TG (22.7 ± 7.3%, *p* < 0.01) and insulin (14.1 ± 10.6%, *p* < 0.05) substantially decreased with training, as HDL-C substantially increased (13.0 ± 1.2%, *p* < 0.01). The results showed no changes in total cholesterol and within-subject variability in LDL-C responses to training was unclear. Although inferences may be limited by the sample size of the control group, this trend seems to be confirmed when individual responses were compared with the control group (Figures 2 and 3). Figures 2 and 3 summarized the comparison between intervention and control group at pre-training, as well as the comparison of pre- and post-training changes between groups.

The results of regression models showed a significant relative contribution of age only for within-subject variability in waist circumference (*b* = -0.48, 90% CL -0.67 to -0.29; *R*^*2*^ = 0.29). The inclusion of age and maturity status in the proportional, curvilinear regression models showed that changes in BMI z-score, waist circumference, HDL-C, TG and insulin were not related to inter-individual variability in maturity status.

## Discussion

The main finding of the present study is that multidisciplinary intervention programs (i.e., aerobic training, nutritional guidance and educational meetings) resulted in beneficial changes in important cardiovascular risk factors, such as TG, HDL-C, insulin, BMI z-score and waist circumference, in a previously sedentary group of obese adolescent girls. To minimize any confounding effects associated with variation in diet, all participants followed a similar diet during the 12-week intervention. The observation suggests that aerobic-based training interventions may result in improved cardiovascular functionality in adolescent girls, with beneficial effects on body composition. Moreover, confounding effects associated with age and maturity status, in metabolic health indicators responses to aerobic-based exercise in obese adolescent girls of contrasting maturity status, appear to be trivial.

Obesity is related to an imbalance between energy input and output, the size of which may be very small if over a long period [24]. Both biological and social factors influence the development and impact of obesogenic behaviors, the consequences of the condition in long-term physical health and the success of certain interventions [25]. In particular, adolescence represents a stage of development when individuals are particularly susceptible to the onset of obesity. With the onset of puberty, adolescents experience marked changes in body size, composition, and physique. Pubertal gains absolute- and relative fat-mass are particularly marked in females, especially those that mature in advance of their peers [11]. The results of the present study add to the data, since it indicates a positive trend of responses in body composition, due to exercise training interventions in obese adolescent girls [4,8,26,27]. Changes in waist circumference may indicate alterations in body shape, which may contribute to the increase of positive self-perception of the body. This is of relevance as adolescence is a period of major physical and behavioral changes [28]. Associated with growth spurt and sexual maturation, adolescent girls need, mainly, to accept their bodies, interact with age peers from both sexes (concern with social acceptance) and strive for independence [29]. Adolescence represents as stage where there are marked changes in a number of health behaviors that are related to weight gain, with adolescents typically becoming less active, more sedentary, more likely to consume fast-food, alcohol and snack foods, and less likely to eat breakfast [30,31]. Future studies should consider possible behavioral changes with exercise based interventions in obese adolescents [25].

It has been reported that moderate intensity of aerobic activities, two or more times a week, lasting at least six weeks, has positive effects in the metabolic profile in the paediatric population [7,8,27,32]. The benefits of physical activity are more evident in obese children and adolescents with previous changes in metabolic profile, such as inadequate lipid profile [7,33]. The characteristics of intensity, frequency and duration of exercise intervention used in the present study are in agreement with previous studies, to induce modifications in metabolic profile [7,8,27,32]. We have found that total cholesterol and LDL-C levels remained unchanged after 12 weeks of physical training and nutritional guidance. There was a substantial decline in the triglyceride level, while HDL-C fasting levels substantially increased with exercise and nutritional guidance. However, such variables did not change in the control group during the 12 weeks (see Figure 2). In the present study, improvements in the lipid profile were observed, since HDL-C levels increased 17% (90% CL 11.9 to 22.3) and TG levels decreased 22% (90% CL 15.1 to 29.5), suggesting possible to likely benefits to metabolic health of adolescent obese girls. These results corroborate with similar interventions in which lipid profile in children and adolescents improves with exercise practice, mainly demonstrated by the increase in HDL-C and the reduction in TG [7,34]. Observation of reduction in total cholesterol and LDL is not always consistent [32,34]. Regular physical exercise may not reduce the levels of total cholesterol and LDL, but result in changes in size and density of LDL subfractions, increasing concentrations of larger LDL and decreasing concentrations of smaller LDL [7,35,36]. Nevertheless, studies analysing subfractions of LDL concentrations in intervention programs in obese populations are sparse. A trend of reduction in LDL-C seems to be apparent when interventions focus mainly on food intervention [37,38].

In this study, changes induced by the intervention were trivial in glucose; however, as presented above, substantial declines in the insulin level were observed in the intervention group. Studies that investigate the relationship between body fat and insulin resistance after exercise have shown different results. Observations based in adolescent obese tend to show reductions in fasting insulin in subjects who participated in intervention group (nutrition and exercise intervention) compared to controls [32,39-41]. In these studies were observed positive changes in body size with interventions. When changes in fasting insulin were not present after intervention programs, no changes in body composition and shape were observed [7]. In the present study, we have observed a possible beneficial decrease in insulin, and similarly with previous observations, with positive changes in body size. However, some studies have evidenced that exercise alone can have a positive impact on insulin resistance risk in obese youth, without changes in body composition [42], probably exercise-induced improvements in insulin metabolism [43]. Exercise-related increases in insulin action is a result of several adaptative mechanisms in muscular, adipose, liver and endothelial tissues [44]. Particularly in girls, physical activity was inversely associated with insulin resistance.

The issue of maturity-associated variation in training responses in obese adolescents has not received much attention. In the present study, we found that only changes induced by the 12-week intervention on waist circumference were related to age. The results indicate that the magnitude of decrease in waist circumference was higher in older girls, in a proportion of approximately 50% to their younger peers. As for menarcheal status influence, the proportional, curvilinear models revealed no influence of the magnitude of responses to training on body size and metabolic health indicators. This conflicts with reports that indicate differences in metabolic health responses with training interventions in children and adolescents of contrasting ages and sexual maturity status, as classified as prepubertal, pubertal and mature [9,10]. However, chronological age varied within and among the three maturity groups and was not statistically controlled in the analysis. This is relevant because maturation should be viewed in two contexts, timing and tempo. Timing refers to when the specific maturational event occurs and tempo refers to the rate at which maturation progresses, and varies considerably among individuals [11]. Particularly, in the present study we used the menarcheal status to assess sexual maturity status, as this is a rather late maturation of puberty that ordinarily occurs after maximum growth in stature [28]. This may explain the trivial differences in stature observed in the subjects, and we can consider that the sample was advanced in maturity status. The need to allow for the independent effects of age may be comprehended if one considers that the average age at menarche for American girls is 12.8 years, although normal variation ranges from 9 through 17 years of age [11]. Menarcheal status is a valid indicator that is based on a maturational event in girls, but it is limited to provide information about tempo, i.e. the rate of at which maturation progresses, in girls that did not attained menarche, thus experiencing most of the changes associated to growth. In future studies, the use of somatic maturation indicators or skeletal age may be more sensitive to reveal possible interactions between changes induced by exercise-based interventions with growth and maturation in obese adolescents.

## Conclusions

In conclusion, the multidisciplinary intervention based on aerobic training used in this study produced substantial benefits to body size and shape (BMI z-score and waist circumference) and to metabolic health indicators (HDL-C, TG and insulin) in overweight girls. Accordingly, the results of the current study suggest that aerobic training, nutritional guidance and educational meetings programs, with 12 weeks of duration, are effective enough to positively influence the metabolic health indicators of overweight girls. Additionally, the observed effects could not be associated with age and maturity status. Future analyses are needed to examine the relation between training responses, growth and maturation, through the observations of somatic maturation and skeletal age.

## Competing interests

The authors declare that they have no competing interests.

## Authors’ contributions

NL participated in the design and coordination of the study. RBR participate in the conception and its design. WAL and GEM contributed in the execution of the study. CP and MJCS contributed substantial in the critical review for important intellectual content. HMC contributed in the statistical analyses and interpretation of data. All authors read and approved the final manuscript.

## Authors’ information

NL researcher and professor at the Department of Physical Education, Federal University of Paraná, Curitiba, Brazil. RBR leader of the Pediatric Endocrinology Unit and professor at the Department of Nutrition, Federal University of Paraná, Curitiba, Brazil. WAL PhD student at the Faculty of Physical Education, State University of Campinas, São Paulo, Brazil. GEM PhD student at the Department of Physical Education, Federal University of Paraná, Curitiba, Brazil. CP researcher and professor at the Department of Anthropology, Research Centre for Anthropology and Health, University of Coimbra, Coimbra, Portugal. HMC researcher and professor at the Faculty of Sports Sciences and Physical Education, University of Coimbra, Coimbra, Portugal. MJCS researcher and professor at the Faculty of Sports Sciences and Physical Education, University of Coimbra, Coimbra, Portugal.
